# Juvenile primary fibromyalgia syndrome: A Review- Treatment and Prognosis

**DOI:** 10.1186/s12969-021-00529-x

**Published:** 2021-05-18

**Authors:** Maya Levy Coles, Yosef Uziel

**Affiliations:** 1 Department of Pediatrics, Meir Medical Center, Pediatric Rheumatology Unit, Kfar Saba, Israel; 2 Sackler School of Medicine, Tel Aviv University, Tel Aviv, Israel

**Keywords:** Fibromyalgia, Juvenile, JPFS, Chronic pain, Musculoskeletal pain syndrome

## Abstract

Juvenile primary fibromyalgia syndrome (JPFS) is a chronic musculoskeletal pain syndrome affecting children and adolescents. In part one of this review, we discussed the epidemiology, etiology, pathogenesis, clinical manifestations and diagnosis of JPFS. Part two focuses on the treatment and prognosis of JPFS. Early intervention is important. The standard of care is multidisciplinary, combining various modalities—most importantly, exercise and cognitive behavioral therapy. Prognosis varies and symptoms may persist into adulthood.

## Background

Chronic musculoskeletal pain is a primary reason for referrals to pediatric rheumatologists [1]. It can be caused by a variety of inflammatory or non-inflammatory conditions, including arthritis, hypermobility, fibromyalgia (FM), growing pains and complex regional pain syndrome (CRPS) Amplified musculoskeletal pain syndrome is a generic, descriptive term used to describe chronic pain syndromes of unconfirmed etiology, such as FM, CRPS and idiopathic musculoskeletal pain. For individuals with this syndrome, pain signals are augmented; thus, mildly painful and nonpainful stimuli are registered by the body as very painful, which leads to attempts to avoid the induction of pain, leading to functional disability.

Our previous review focused on CRPS [2]. Yunus and Masi were the first to describe and use the term juvenile primary fibromyalgia syndrome (JPFS), in a clinical study published in 1985, in which they suggested diagnostic criteria based on 33 juveniles ages 17 years or younger, who suffered from chronic pain.

JPFS is defined by chronic diffuse musculoskeletal aching and pain, which is the hallmark of this condition, and multiple predictable tender points. Part one of this review summarized the current information regarding the epidemiology, etiology, clinical manifestations and diagnosis of JPFS [3]. This part of the review focuses on treatment and prognosis.

## Treatment

The primary goal in the treatment of JPFS is to enhance quality of life through pain relief and improved function. A multidisciplinary approach, combining behavioral and exercise-based modalities is currently the standard of care for JPFS [4, 5]. Treatment should begin with non-pharmacologic modalities, most importantly exercise. Other non-pharmacologic modalities include movement and meditative treatments, and Cognitive Behavioral Therapy (CBT) [6]. Reassurance is very important, including acknowledging that the patient’s pain is real, and emphasizing that it is not dangerous. Caregivers should communicate that although the pain may last for an unknown period and no immediate cure exists, there are therapeutic options that may reduce the pain over time. Setting goals of leading a full life, stressing the importance of lifestyle factors and treating emotional symptoms are critical.

Multidisciplinary programs may be implemented in a hospital or outpatient setting. In a study comparing an intensive day-hospital rehabilitation program of physical, occupational and psychological therapies to outpatient treatment for children with significant pain-related disability, those enrolled in the day-hospital rehabilitation had significantly larger improvements in functional disability, pain-related fear, and readiness to change [7]. Intensive, multidisciplinary outpatient programs have also reported improved pain, function, and quality of life in children with JPFS [8].

A recent article suggested that the heterogeneity of the clinical presentation associated with FM, and the modest results, on average, for any therapy, call for a more individualized management strategy [9]. For example, The European League Against Rheumatism guidelines recommend a tailored approach directed by key FM symptoms of pain, sleep disorders, fatigue, functional disability [10].

### Physical therapy

Exercise and physical therapy are main cornerstones in the treatment of all JPFS patients [11]. Benefits of exercise, which include mainly aerobic training, as well as strength/resistance training or movement-based therapies such as yoga and tai chi, have primarily been demonstrated in studies of FM treatment in adults [12–16]. Decreased pain and fatigue, and improvement in daily function and quality of life were noted.

The American Pain Society guidelines for children with FM recommend at least 30 min of moderate-to-vigorous cardiovascular exercise two to 3 days a week [17].

Despite recommendations for exercise to manage juvenile FM pain, long-term exercise adherence in patients with FM is poor [18]. It has been demonstrated via actigraphy, that children with chronic pain are less physically active than their peers are [19]. Neurobiological alterations explain why exercise may be associated with increased short-term pain among patients with FM. However, various studies suggest that neurobiological changes associated with regular exercise have the potential to lead to long-term pain reduction, if FM patients can overcome the short-term increases in pain after exercise and avoid the increased fear of movement that discourages them from engaging in physical activity [20]. Most studies on exercise for FM are relatively short-term. Additional studies on long-term exercise programs are needed to determine whether adherence to exercise, as well as its benefits can be maintained over time.

A 2015 study by Sil et al. showed preliminary evidence of objective functional deficits in movement biomechanics (affecting gait, posture, balance, and movement), among adolescents with JPFS, as compared to their peers. These biomechanical alterations make them more prone to pain or injury during exercise, and may reinforce their activity avoidance [18].

Few exercise approaches and programs for the treatment of juvenile FM have been studied. In a small-scale, randomized, controlled trial of an exercise intervention in children with FM, Stephens et al. evaluated the feasibility and effectiveness of a 12-week aerobic exercise program versus qigong [21]. Both groups experienced improvement in FM symptoms and pain measures, but children in the aerobic training program exhibited greater improvements in fatigue, quality of life and physical functioning.

An interventional study investigated whether multidisciplinary treatment, including intensive exercise therapy would improve sleep quality in adolescents with JPFS. Although perceived sleep quality improved, objective measures did not [22].

Combined CBT and exercise-based interventions are often used clinically as part of multidisciplinary pain treatment programs. Despite its effectiveness in improving daily functioning and in coping with pain in adolescents with JPFS [23, 24], CBT did not independently result in increased physical activity, as was shown in a clinical trial via actigraphy monitoring [25]. Therefore, a multidisciplinary approach combining CBT and additional interventions to increase physical therapy is recommended.

An intervention combining CBT techniques with neuromuscular training: Fibromyalgia Integrative Training for Teens (FIT Teens), showed initial promise in adolescents with JPFS [26, 27]. Neuromuscular training, modified from evidence-based injury prevention protocols in pediatric sports medicine, is a tailored approach that targets biomechanical deficits in gait, posture, balance and movement, to reduce risks for injury or pain among patients with JPFS. Therefore, biomechanical assessment can be used to objectively demonstrate observable changes in performance after such an intervention [28]. A recent randomized, controlled, pilot study provided preliminary evidence that the FIT Teens intervention offers stronger treatment benefits than does CBT alone, particularly in reducing pain [29]. No additional randomized controlled trials of exercise interventions in juvenile FM were found.

It has been proposed that less strenuous forms of self-paced physical activity and lifestyle modifications may lead to greater long-term adherence [30]. A recent study of adult women with FM demonstrated that substituting sedentary time with light or moderate-to-vigorous physical activity was associated with better quality of life and lower disease impact [31]. However, in a “Lifestyle Physical Activity” approach study in adults with FM, that consisted of strategies to increase incidental exercise throughout the day, beneficial effects were not sustained over time [32]. No similar studies using this approach were found in juvenile FM.

### Psychological therapy

Psychological therapies, most prominently CBT), are the strongest evidence-based treatment modalities for JPFS. They are recommended as an integral part of the interdisciplinary treatment approach [4].

CBT techniques offer short-term, goal-oriented psychotherapy, emphasizing changes in thought patterns and behaviors. CBT has been found to be effective in reducing functional disability and in improving the ability to cope with pain among adolescents with JPFS.

An analysis of the results of 13 JPFS treatment programs using CBT alone or in combination with other treatment modalities, concluded that it provided worthwhile improvements in pain-related behavior, self-efficacy, coping strategies and overall physical function [33]. Sustained improvements in pain were most evident when individualized CBT was used.

Treatment programs for chronic pain typically take a rehabilitative approach. The initial emphasis is on decreasing pain-related disability, with the expectation that pain reduction will follow. A study that examined this hypothesis in youth with chronic pain, demonstrated a significant, rapid reduction in functional disability, as compared to pain reduction following CBT among youth with chronic pain [34].

In a multisite, single-blind, randomized, controlled clinical trial, CBT was compared to FM education for treating juvenile FM [23]. Patients (114 adolescents) in both groups had significant reductions in functional disability, pain, and symptoms of depression, but CBT was significantly superior to FM education in reducing functional disability. A subsequent study aimed to identify predictors for treatment response (reducing functional disability) at the 6-month follow-up, in the above clinical trial. Patients with greater initial functional disability and higher coping self-efficacy were significantly more likely to achieve a clinically significant improvement in functioning after CBT. Pain intensity, depressive symptoms, and parental pain history did not significantly predict treatment response [35].

The therapist is by necessity unblinded to the treatment as are the patients, who likely know when they are being treated with CBT. There is no such thing as a “placebo” for behavioral treatment. Hence, behavioral trials are usually “single-blind” studies in which the assessors are blinded and control groups are often “Attention-control” or “Education” comparison groups in which the active ingredients of treatment (e.g., CBT) are not delivered.

The effectiveness of psychotherapy has been demonstrated in many studies of pediatric patients with chronic pain. A 2018 Cochrane review, discussing psychological therapies for the management of chronic and recurrent pediatric pain [36], concluded that they might be effective for reducing pain frequency and intensity in children and adolescents with chronic pain conditions post-treatment, but not at follow-up. Psychological therapies were beneficial for reducing disability in children with mixed chronic pain conditions at post-treatment and at follow-up, and for children with headache at follow-up. No beneficial effect was found for improving depression or anxiety.

To address barriers to treatment access, such as distance and costs, technology (such as the Internet, computer-based programs, and smartphone applications) is being used to deliver psychological therapies remotely to children and adolescents with chronic pain. A 2019 Cochrane review [37] examined the efficacy of remotely-delivered psychological therapies for the management of chronic pediatric pain. They were found to reduce headache severity post-treatment. For the remaining outcomes, either no beneficial effect was found at post-treatment or follow-up, or evidence to determine an effect was lacking. Overall, participant satisfaction with treatment was positive. However, the quality of evidence was very low, and further studies are needed in this potentially promising field.

Studies have suggested a role or coping with pain and aspects of cognitive appraisal (psychological perceptions of pain) as potential psychological changes that explain CBT-related improvements among youth with juvenile FM [38]. Kashikar-Zuck et al. examined the psychological processes of CBT effectiveness in adolescents with juvenile FM [24]. CBT led to significant improvements in pain coping, catastrophizing, and coping efficacy, which were sustained over time. However, it was not found to mediate improvement in functional disability or depressive symptoms from post-treatment to 6-month follow-up.

A significant number of FM patients suffer from co-morbid psychiatric disorders, mainly anxiety and depression, and may benefit from referral to a psychiatrist. However, a subset of FM patients demonstrate strong emotional resilience despite their chronic symptoms and do not need psychiatric follow-up [6, 39].

### Pharmacological treatments

Currently, evidence for the efficacy of pharmaceuticals for the treatment of JPFS is limited. While several medications are approved by the US Food and Drug Administration (FDA) for the treatment of adults with FM (duloxetine, milnacipran and pregabalin), there are none for the treatment of juvenile FM [40, 41]. To our knowledge, no large-scale clinical trials of medications for JPFS are being conducted. Additional stringent, randomized controlled trials with longer follow-up periods are needed to determine the long-term efficacy and safety of medications for treating JPFS. Management of JPFS should include an emphasis on non-pharmacological approaches, although judicial use of medications may be considered for symptom management.

#### Non-opioid analgesics and anti-inflammatory medications

Topical analgesics, as well as oral over-the-counter analgesics, such as acetaminophen and non-steroidal anti-inflammatory drugs, have been used to treat adult and juvenile FM, but they are not effective [40, 42]. This is hypothesized to be due to their peripheral action, while the underlying pain mechanism in FM is centrally mediated [43]. Prednisone has not been found effective for adult FM [44].

#### Anticonvulsants

The gabapentinoids were originally used as anti-epileptics, but are commonly prescribed for chronic pain. Studies of pregabalin and gabapentin for treatment of adult FM have demonstrated efficacy and good tolerability [45]. Pregabalin is FDA-approved for the treatment of adult FM [41] and is recommended in treatment guidelines for adult FM [10, 46, 47]. A single-center study that examined the potential benefit of a novel form of extended-release gabapentin for adult FM [48] demonstrated significant pain relief, but was limited by small sample size, short treatment duration (15 weeks) and lack of a control group. There is a paucity of evidence for the analgesic effect and safety of gabapentin and pregabalin in children and adolescents [49]. A double-blind, randomized, placebo-controlled trial from 2016 that examined the safety and efficacy of pregabalin in adolescents with FM, did not demonstrate clear efficacy, and had a slightly worse safety profile, as compared with adults [50].

#### Anti-depressants

Three main classes of anti-depressants are employed in the treatment of adult FM, with limited data supporting their use in the pediatric population. These include serotonin-norepinephrine reuptake inhibitors (SNRIs), Selective serotonin reuptake inhibitors (SSRIs), and tricyclic antidepressants.

##### SNRIs

The two main SNRIs, duloxetine and milnacipran, are approved by the FDA, but not by the European Medical Agencies for the treatment of adult FM [51]. Current evidence on SNRIs for the treatment of FM in adults [51] suggests that duloxetine and milnacipran may provide a clinically relevant benefit over placebo in the frequency of pain relief. Limited data exist in the pediatric population. In a recent study in adolescents with JPFS, significantly more patients on duloxetine compared to placebo experienced decreased pain severity and there were no safety concerns [52]. Milnacipran was also assessed in JPFS [53]. The open-label phase of the study demonstrated improvement in pain and quality of life among those treated with milnacipran and it was well-tolerated. However, the study was terminated early due to low enrollment rates.

##### SSRIs

Selective serotonin reuptake inhibitors (SSRIs) such as fluoxetine and paroxetine have had less convincing evidence for efficacy in adults with FM [54, 55]. An exploratory, open-trial study of fluoxetine in juvenile FM demonstrated reduced pain and global improvement, but only low doses of the medication were tolerated, suggesting increased sensitivity of children to adverse effects [56].

##### Tricyclic antidepressants

Amitriptyline is the tricyclic antidepressant prescribed for FM, based on studies limited to the adult population. In a randomized controlled trial that compared the effectiveness of naproxen versus amitriptyline for adults with FM, the latter was found to have a positive effect on various outcomes [42]. In a review from 2008, a beneficial therapeutic response was demonstrated after 6 to 8 weeks of treatment with 25 mg/day amitriptyline, but no effect was demonstrated when treated with a higher dose or for longer duration [57]. A systematic review from 2011 that compared the efficacy of amitriptyline, duloxetine and milnacipran, concluded that amitriptyline was superior in reducing pain, sleep disturbances and fatigue [58]. However, the validity of these findings is limited by low methodological quality and inconsistencies in data regarding adverse effects.

While there is little evidence supporting the use of anti-depressants for the treatment of JPFS, they have an important role in treating concomitant psychiatric conditions, such as anxiety and depression, that are common among children with FM [59, 60]. The black box warning for increased suicidal tendency in young adults with major depressive disorder taking SSRIs should be carefully considered [61].

#### Opioid analgesics

Opioid analgesics are not effective for adult FM [62] and are not recommended in current adult FM treatment guidelines. Despite lack of evidence regarding the use of opioids for treating juvenile FM, opioid prescription for children doubled between 1990 and 2010 [63]; an alarming trend associated with adverse consequences. Accidental opioid poisoning in young children may lead to disability or death [64]. Use of opioid analgesics should be avoided in the treatment of JPFS, also due to the potential for dependency and abuse.

#### Opiate receptor antagonists

Studies have shown that low-dose naltrexone, an opiate receptor antagonist, may be effective, safe and inexpensive for adults FM [65]; yet, further large prospective controlled trials are needed before its use can be recommended for JPFS [66]. To our knowledge, no studies have been published regarding its use for the treatment of JPFS.

#### Muscle relaxants

Cyclobenzaprine, a muscle relaxant structurally similar to tricyclic antidepressants, is weakly recommended for treatment of FM in adults with sleep disturbances [10]. Further research is needed regarding the efficacy of a sublingual formulation of low-dose (2.8 mg), slow-release cyclobenzaprine, which may be better suited for children due to its decreased frequency of administration and non-tablet formulation [41].

#### Medical Cannabis treatment

Cannabinoids may be useful in the management of FM due to their effects on pain and associated symptoms [67]. Recent studies indicate a possible clinical advantage and safety of adjunctive medical cannabis treatment in adult FM patients [68]. In a prospective, observational study that assessed the effects of adding medical cannabis to the standard analgesic treatment of 102 adult FM patients [69], a significant improvement in FM outcome severity scales was observed in approximately 40%, especially in those with sleep dysfunctions. A moderate improvement in the anxiety and depression scales was observed in 50% of patients, and one-third experienced mild adverse events. However, available evidence on medical cannabis in FM is very scarce, and no clinical trials were found regarding its use in pediatric FM patients. Further studies are needed to confirm current findings and to assess the efficacy and safety of medical cannabis in the pediatric population.

### Complementary medicine

A significant number of adult patients use complementary treatments to relieve stress, pain, physical and psychological impairment [47], most of which have never been tried in children. Modalities include herbs, lotions, multivitamins, dietary interventions and mind-body interventions [70].

#### Dietary interventions

Various studies suggest an association between vitamin D deficiency and FM in adults, and a possible effect of vitamin D supplementation therapy in improving musculoskeletal symptoms, depression, and quality of life [71–73]. However, the causal relationship is inconclusive and conflicting results were obtained regarding the effect of vitamin D supplementation on symptom control. In a 2016 pilot study, children with musculoskeletal and orthopedic conditions, chronic or recurrent pain, and vitamin D deficiency were prescribed vitamin D replacement therapy for 6 months, with improvement in pain intensity and daily functioning [74]. Additional large-scale randomized studies with JPFS patients are needed to validate these findings. Vitamin D supplementation may be considered a co-adjuvant in JPFS therapy.

#### Mind-body interventions

A systematic review of complementary and alternative medicine for treating FM pain, concluded that balneotherapy and mind-body therapies showed the most promising findings [75].

Mindfulness meditation may affect pain variables in adolescents through its relation to pain catastrophizing [76]. A randomized, controlled pilot study of mindfulness-based stress reduction for pediatric chronic pain, suggested increased mindfulness was effective, but showed inconsistent patterns with other outcome measures [77]. Self-reported outcomes after a mindfulness-based stress reduction intervention that included mindfulness and yoga for adults with FM, suggested potential benefits, but objective cardiac autonomic parameters did not [78, 79].

Randomized controlled trials of Tai Chi mind-body treatment for adults with FM demonstrated improvement in symptoms [80], and concluded that Tai Chi resulted in similar or greater improvement in various symptoms, as compared with aerobic exercise [81]. This mind-body approach may be considered a therapeutic option in the multidisciplinary management of FM. Further research on this modality for children and adolescents is needed.

#### Acupuncture

Acupuncture can be an effective adjuvant in the care of pediatric patients with chronic pain conditions, and may be clinically valuable in a multidisciplinary treatment program [82]. Studies of acupuncture in adults with FM exhibit conflicting results [75, 83, 84]. Therefore, additional studies are needed before this modality can be recommended for JPFS treatment.

#### Guided imagery and hypnosis

A systematic review and meta-analysis on guided imagery and hypnosis for fibromyalgia in adults [85], found it had a clinically relevant benefit in the outcomes of pain relief and psychological distress at the end of therapy, as compared with controls. Combined hypnosis with CBT was superior to CBT alone in reducing psychological distress at the end of therapy, but not in other outcomes.

### Nerve stimulation

Transcutaneous Electrical Nerve Stimulation (TENS) is a noninvasive therapeutic method that uses low-voltage electrical current for pain relief, by activating endogenous pain inhibitory mechanisms [86]. The use of this modality for adult FM has been described in the literature [87]. It demonstrated significant improvements in movement-evoked pain, fatigue and other clinical outcomes [88]. There are currently no prospective, blinded studies of TENS use in juvenile FM, and further studies are needed to provide evidence of its efficacy in the pediatric population.

Studies suggest that non-invasive brain stimulation techniques, including repetitive transcranial magnetic stimulation and transcranial direct current stimulation, may be feasible and safe modalities as add-on treatments for adult FM [89]. They reduced pain levels and fatigue and improved daily functioning, with no serious adverse effects [90]. However, additional, substantially larger, studies of this modality are needed before applying it to children.

### Additional therapies

Additional therapies studied in adults with FM include hyperbaric oxygen therapy [91, 92], occupational therapy [93] and climatotherapy [94]. They have not yet been studied in the pediatric population.

## Prognosis

JPFS is likely to be a long-term condition for many patients. Initial studies indicated a positive prognosis, with improvement or resolution of symptoms over one to several years of follow-up [95–97]. However, more recent studies indicated a less favorable prognosis, demonstrating that physical and psychosocial symptoms of FM appear to be chronic in many patients [98, 99].

In a study by Connelly et al., children with JPFS exhibited worsening pain and quality of life over time, regardless of treatment modality or patient compliance with therapy [100]. In a follow-up study on long-term outcomes of youth with JPFS compared to healthy controls, 62.5% of participants in the JPFS group continued to experience widespread pain and 60.4% reported having all the cardinal features of FM at follow-up (range 2–6 years) [101]. In a similar prospective study, over 80% of patients continued to experience FM symptoms in early adulthood and 51.1% of the JPFS patients met the adult American College of Rheumatology criteria for FM [102]. Interestingly, in addition to physical and emotional impairment, the JPFS group did not achieve similar levels of education and fewer were married as compared to healthy control subjects of similar ages. In a summary of data on youth with JPFS, enrolled in the Childhood Arthritis and Rheumatology Research Alliance Legacy Registry, indicators of function and well-being were found to either worsen over time or remain relatively unchanged [103].

The effect of age at onset on prognosis is unclear. A study compared characteristics of children with JPFS onset at age 10 years or younger versus onset after age 10. After a mean follow-up of 14 months, there was no difference in outcomes between the groups [104]. However, since better outcomes were exhibited in children compared to adults, early detection of FM may indicate better prognosis [105].

While symptoms may be chronic, their severity may improve over time. In a recent prospective study [106], Kashikar-Zuck et al. examined longitudinal trajectories of pain and depressive symptoms in JPFS patients from adolescence to young adulthood, and the impact of these symptoms on physical functioning over time. At the 8-year follow-up (mean age 24.2 years), most continued to suffer from pain and psychological impairment. However, steady improvement or rapid-rebounding improvement in pain severity was observed over time. Depressive symptoms improved, remained low-stable, or worsened over time, with the latter subgroup associated with poorer physical functioning over time. Consequently, JPFS patients with worsening depressive symptoms may require more intensive interventions to prevent long-term disability. The significant association of these symptoms with school absence, a risk factor for long-term consequences [107], including various psychiatric, economic, social and marital problems in adulthood [108] emphasizes the importance of addressing depressive and other psychiatric symptoms in adolescents with JPFS as well [59].

Cognitive factors such as catastrophizing and fear of movement have been shown to be poor prognostic factors in chronic pain conditions, including juvenile FM [24].

Family environment may affect JPFS prognosis. A controlled 4-year, follow-up study on the impact of family environment on the long-term adjustment of patients with JPFS, indicated that adolescents from controlling family environments are at increased risk for poorer emotional functioning in early adulthood [109]. Another study indicated low levels of resilience to non-inflammatory chronic musculoskeletal pain syndrome in adolescent patients and their parents, with a negative effect on symptoms [110]. Therefore, behavioral and family interventions should foster independent coping among adolescents with JPFS and greater parental flexibility, to enhance successful long-term emotional functioning. Social support is a predictor of function and symptoms in adolescents with JPFS, as well [111]. Medical caregivers may experience difficulties and frustration when dealing with teenage patients who remain dysfunctional over time, despite their interventions.

Additional prospective research is needed to determine the long-term prognosis of juvenile FM patients and implications for treatment.

## Conclusions

Juvenile primary FM syndrome is a chronic, musculoskeletal pain syndrome. Standard care consists of a multidisciplinary approach. Physical exercise in conjunction with psychological treatment comprise the mainstay of therapy. Management should rely on non-pharmacological approaches, but judicial use of medications may be considered for symptom management. The treatment plan should be individually tailored, taking into consideration the child’s environment, including familial, social and academic components. Data regarding the prognosis of JPFS conflict, and it may be a long-term condition for many patients. Additional high-quality studies are needed to deepen understanding of etiologic factors, develop better diagnostic tools, and verify effective treatment programs for the pediatric population, with the goals of early detection, improved treatment outcomes and better long-term prognosis.

## Data Availability

Data sharing is not applicable to this article as no datasets were generated or analyzed during the current study.

## Abbreviations

CBT: Cognitive behavioral therapy
CRPS: Complex regional pain syndrome
FDA: United States. food and drug administration
FIT Teens: Fibromyalgia integrative training for teens
FM: Fibromyalgia
JPFS: Juvenile primary fibromyalgia syndrome
SNRIs: Serotonin norepinephrine reuptake inhibitors
SSRIs: Selective serotonin reuptake inhibitors
TENS: Transcutaneous electrical nerve stimulation
